# Association between cerebral dopamine neurotrophic factor (*CDNF*) 2 polymorphisms and schizophrenia susceptibility and symptoms in the Han Chinese population

**DOI:** 10.1186/s12993-017-0133-4

**Published:** 2018-01-03

**Authors:** Yongfeng Yang, Hongyan Yu, Wenqiang Li, Bing Liu, Hongxing Zhang, Shuang Ding, Yanli Lu, Tianzi Jiang, Luxian Lv

**Affiliations:** 1 Key Laboratory for NeuroInformation of Ministry of Education, School of Life Science and Technology, University of Electronic Science and Technology of China, Chengdu, China; 2 Department of Psychiatry, Henan Mental Hospital, The Second Affiliated Hospital of Xinxiang Medical University, Xinxiang, China; 3 Henan Key Lab of Biological Psychiatry, Xinxiang Medical University, Xinxiang, China; 4 Brainnetome Center, Institute of Automation, Chinese Academy of Sciences, Beijing, 100190 China; 5 National Laboratory of Pattern Recognition, Institute of Automation, Chinese Academy of Sciences, Beijing, 100190 China; 6 The Queensland Brain Institute, University of Queensland, Brisbane, QLD 4072 Australia; 7 Department of Psychiatry of the Second Affiliated Hospital of Xinxiang Medical University, No. 388, Jianshe Middle Road, Xinxiang, 453002 China

**Keywords:** *CDNF2*, Polymorphism, PANSS, Schizophrenia

## Abstract

**Background:**

Schizophrenia (SZ) is a complex polygenic psychiatric disorder caused in part by abnormal dopamine levels. Cerebral dopamine neurotrophic factor (*CDNF*) 2 is known to protect and repair the dopaminergic system. Dopamine dysfunction is one of the pathogenesis of SZ. However, the relationship between *CDNF2* and SZ has not been previously investigated. We speculated that *CDNF2* may be a susceptibility factor for SZ.

**Methods:**

To address this issue, we carried out a study to investigate the association between *CDNF2* and SZ in the total sample 1371 (670 SZ patients and 701 healthy controls) Han Chinese population. Stage 1 included 528 SZ patients and 528 healthy controls; and stage 2 included 142 SZ patients and 173 healthy controls. The allele and genotype frequencies of five single nucleotide polymorphisms (rs2577074, rs2577075, rs2249810, rs6506891, and rs2118343) of *CDNF2* were compared between patients and controls.

**Results:**

We found a significant association in allele and genotype frequencies between the two groups at rs2249810 (χ^2^ = 4.38 and 6.45, respectively; *P* = 0.03 and 0.04, respectively). An association was also observed in males at rs2249810 (χ^2^ = 8.76; *P* = 0.03). Haplotype TGATC differed between SZ and controls in stage 2 samples (χ^2^ = 6.38; *P* = 0.01), and rs2118343 genotypes were associated with negative factor scores (F = 4.396; *P* = 0.01).

**Conclusions:**

These results suggest that rs2249810 and haplotype TGATC of *CDNF2* are an SZ susceptibility locus and factor, respectively, and that rs2118343 genotypes are associated with negative symptoms of SZ in the Han Chinese population.

## Background

Schizophrenia (SZ) is a complex psychiatric disorder with an estimated heritability of 60–80% that affects 1.0% of the global population [1–3]. Previous studies have reported that the absolute risk of suicide in SZ to be 6.55% in male and 4.91% in female [4], and suicide was 13-fold higher for SZ than the general population [5]. The core symptoms of SZ vary in terms of severity among patients [6]. Research on the genetic basis of SZ has focused on identifying polymorphisms in candidate genes and linkage regions [7–10]. To date, 108 SZ-associated gene loci have been identified in a genome-wide association analysis (GWAS) of the largest sample size to date (36,989 cases vs. 113,075 controls) [11]. However, others have reported that multiple genes contribute weakly or moderately to SZ pathogenesis [12]. Identifying SZ susceptibility genes among numerous candidates is an ongoing challenge.

Abnormal dopamine levels underlie SZ. Neurotrophic factors, such as brain-derived neurotrophic factor (*BDNF*), nerve growth factor, glial cell line-derived neurotrophic factor, and cerebral dopamine neurotrophic factor (*CDNF*), can repair and protect the dopaminergic system. Previous studies have shown that *BDNF* is involved in the pathophysiology of SZ, with *BDNF* levels reduced in the serum and cerebrospinal fluid of patients [13]. *CDNF* has been shown to protect and repair the dopaminergic system in rat models of Parkinson’s disease (PD) [14], which is characterized by degeneration of dopaminergic neurons; it may thus have therapeutic benefits [15]. However, the relationship between *CDNF* and SZ has not been previously investigated. We speculated that *CDNF* is a susceptibility factor for SZ. We tested this hypothesis by examining *CDNF* single nucleotide polymorphisms (SNPs) in two study stages with independent sample sets.

## Methods

Stage 1 samples were those from our previous publications [16, 17], and included 528 paranoid SZ patients (mean age: 27.32 ± 8.03 years old) and 528 healthy controls (mean age: 27.73 ± 8.01 years old) who were recruited from March 2005 to December 2008. Stage 2 samples included 142 SZ patients (paranoid, n = 122 and undifferentiated, n = 20; mean age: 29.28 ± 7.17 years old) and 173 healthy controls (mean age: 32.49 ± 7.43 years old) who were recruited from May 2011 to December 2014.

SZ was diagnosed as previously described [16, 17] according to the criteria listed in the Diagnostic and Statistical Manual of Mental Disorders Fourth Edition (DSM-IV). Exclusion criteria were as follows: patients had been diagnosed with other psychiatric disorders; or had organic brain disease, substance dependence, severe medical complications, or neurological diseases. Family mental health history (FH) was defined as at least one first- or second-degree relative of the proband who met DSM-IV criteria for SZ or schizoaffective disorder. The Positive and Negative Symptom Scale (PANSS) was used to evaluate psychotic syndromes. Five factors were derived from the PANSS, including positive symptoms, negative symptoms, cognition, expression/anxiety, and excitement/hostility [18].

A total of 372 SZ patients (stage 1, n = 229 and stage 2, n = 143) who were not taking antipsychotic medications were evaluated for psychotic syndromes using the PANSS [19]. Inclusion and exclusion criteria for healthy controls were as described our previous papers [16, 17]. These subjects were screened by psychiatrists in simple non-structured interviews. All participants were unrelated Han Chinese who were born and living in North Henan province.

Five SNPs were selected as described in our previous work [16, 17] and covered the 26974145–26995906 genomic intron region on chromosome 10. In stage 1, genotyping was carried out as detailed in our earlier studies [16, 17] using Illumina GoldenGate assays on a BeadStation 500G Genotyping System (Illumina, San Diego, CA, USA). In stage 2, samples were genotyped using a standard Illumina genotyping protocol.

Statistical analyses in this study were performed as described in our previous papers [16, 17]. Power analyses were performed using the G*Power software to calculated (http://www.gpower.hhu.de/) for this study [20, 21]. Genotype and allele frequencies were analyzed using Haploview v.4.1. Hardy–Weinberg equilibrium was assessed with the χ^2^ test with one degree of freedom. Associations between the five factors from the PANSS and different genotype carriers were evaluated by analysis of variance. Bonferroni correction for multiple pair-wise comparisons was conducted for the X × phenotype interaction to reduce the probability of false positives. *P* < 0.05 was considered statistically significant. The corrected α′ (P = 0.01) is α (P = 0.05) divided by the number of possible comparisons.

## Results

To identify allelic variants of the *CDNF2* gene that are associated with SZ, we analyzed two sets of samples. The sampling success rate for subjects and SNPs was 99.84%. Power analyses revealed that the total sample size (n = 1371) had a power of 0.96 to detect a small effect (r = 0.1–0.23), and a power of 1.00 to detect both medium (r = 0.24–0.36) and large (r > 0.37) effects on genotype distributions. For allele frequency, the sample size (n = 2742) had the power (0.91–1.00) to detect small, medium, and large effects.

**Fig. 1:**
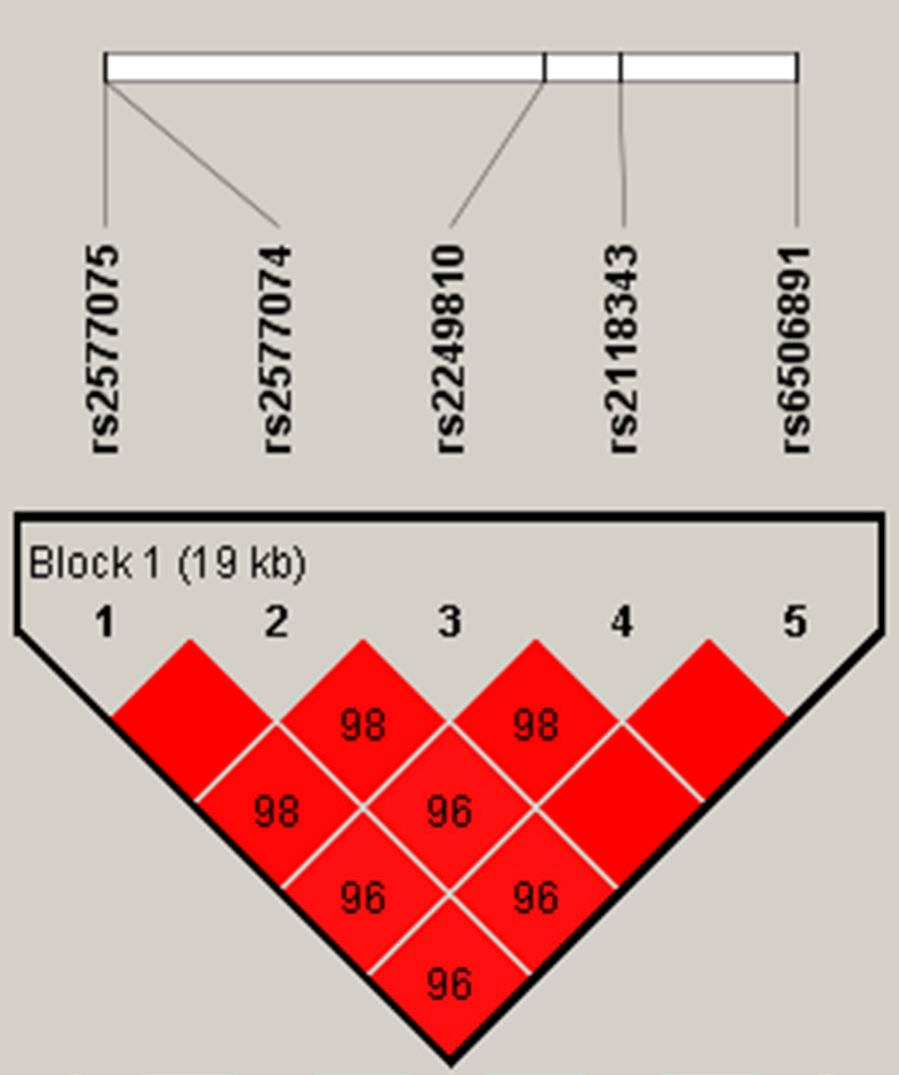
Haplotype block structure of the *CDNF2* gene in both SZ patients and healthy controls in stage 2 samples. The index association SNP is represented by a diamond. The colors of the remaining SNPs (circles) indicate LD with the index SNP based on pairwise r^2^ values from our data

None of the genotype distributions of the other four SNPs significantly deviated from HWE except for SNP rs6506891. There were no significant differences in genotype and allele frequencies between SZ and controls at five SNPs in stage 1 samples (*P* > 0.05, Table 1), even after subdividing by gender and FH (*P* > 0.05). However, there were significant differences in genotype and allele frequencies between SZ and controls at rs2249810, rs6506891, and rs2118343 in stage 2 samples. We also noted differences at rs2249810 when stage 1 and 2 samples were combined. This association was present at rs2249810 in males (χ^2^ = 8.76; *P* = 0.03). To further analyze haplotype structures in this sample, we evaluated pairwise linkage disequilibrium (LD) of five SNPs in SZ patients and controls using standardized D′ and r^2^ values. Haplotypes were identified at five SNPs of *CDNF2* in stage 1 and 2 samples. The positions of these SNPs in stage 2, LD structure, and D′ values for all variants are shown in Fig. 1. Five SNPs formed one LD block, yielding four haplotypes; one of these (TGATC) differed significantly between SZ and controls only in stage 2 samples (χ^2^ = 6.38; *P* = 0.01) (Table 2).

**Table 1:** Genotype and allele frequencies of SNPs in the *CDNF2* gene in patients with SZ and controls

dbSNP ID	Stage	Allele (D/d)^a^	Patients						Controls						*P* value^c^		Combine *P* value	
			n^b^	HWE (*p*)	Genotype			MAF	n^b^	HWE (*P*)	Genotype			MAF				
					DD	Dd	dd				DD	Dd	dd		Genotype	Allele	Genotype	Allele
rs2577074	1	G/A	528	0.35	144	253	131	0.49	528	0.12	140	281	107	0.469	0.14	0.09	0.43	0.89
	2		142	0.58	44	73	25	0.18	173	0.32	41	93	39	0.225	0.28	0.13		
rs2577075	1	A/G	527	0.37	143	253	131	0.49	527	0.13	140	280	107	0.469	0.15	0.29	0.15	0.14
	2		142	0.58	44	73	25	0.18	170	0.22	40	93	37	0.218	0.30	0.15		
rs2249810	1	G/A	528	0.18	186	242	100	0.42	528	0.43	189	261	78	0.395	0.18	0.38	*0.04*	*0.03*
	2		139	0.83	63	62	14	0.10	172	0.65	59	81	32	0.186	*0.04*	*0.01*		
rs6506891	1	T/A	526	*0.00*	285	143	98	0.32	526	*0.00*	299	151	76	0.288	0.19	0.38	0.78	0.41
	2		142	0.93	64	63	15	0.11	173	0.81	58	83	32	0.185	*0.04*	*0.01*		
rs2118343	1	C/G	528	0.83	309	191	28	0.23	526	0.32	318	187	21	0.218	0.56	0.41	0.76	0.45
	2		142	0.53	97	42	3	0.02	173	0.18	101	58	14	0.081	*0.03*	*0.02*		

Significant values has been emphasized by italic

^a^Major/minor allele, major and minor alleles are denoted by D and d, respectively

^b^Number of samples which are well genotyped

^c^*P* values in the parenthesis were analyzed with 10,000 random permutations

**Table 2:** Haplotypes of *CDNF2* in stage 2 patients with SZ

Haplotype	Case (freq)	Control (freq)	Chi square	*P* value	Odds ratio (95% CI)
CAGAG	157.87 (0.57)	169.89 (0.50)	2.25	0.13	1.29 (0.94–1.78)
TGATC	45.87 (0.17)	82.00 (0.24)	6.38	*0.01*	0.61 (0.41–0.92)
TGATG	43.00 (0.16)	57.90 (0.17)	0.30	0.58	0.88 (0.57–1.35)
TGGAG	30.13 (0.11)	26.11 (0.08)	2.61	0.11	1.44 (0.83–2.51)

Significant value has been emphasized by italic

*CI* confidence interval

To explore the association between *CDNF2* variations and SZ symptoms, 371 first-onset SZ patients (stage 1, n = 228 and stage 2, n = 143) with complete PANSS scores were selected from stages 1 and 2. Only rs2577075 genotypes were associated with cognition factor scores in stage 2 samples after Bonferroni correction (F = 3.39; *P* = 0.03) (Table 3). We also only found rs2577075 genotypes were associated with cognition factor scores in female when subdivided by gender after Bonferroni correction (F = 4.05; *P* = 0.04). Other SNPs from stage 1 and 2 samples did not show any associations (*P* > 0.05). However, rs2118343 genotypes were significantly associated with negative factor scores when stage 1 and 2 samples were combined (F = 4.396, *P* = 0.01). We found rs2118343 genotypes were associated with depression/anxiety and excitement/hostility scores in female when subdivided by gender in combined samples after Bonferroni correction (F = 4.39, 4.73; *P* = 0.04, 0.03, respectively).

**Table 3:** Association analysis between five factors of PANSS and five SNPs of *CDNF2* in patients with SZ

SNP	Genotype	Total PANSS		Positive		Negative		Depression/anxiety		Cognition		Excitement/hostility	
		Stage 1	Stage 2	Stage 1	Stage 2	Stage 1	Stage 2	Stage 1	Stage 2	Stage 1	Stage 2	Stage 1	Stage 2
rs2577075	AA	80.46 ± 18.15	86.36 ± 8.49	20.22 ± 6.60	22.63 ± 5.53	17.02 ± 5.45	19.70 ± 3.39	14.39 ± 2.97	14.51 ± 2.28	16.73 ± 5.62	17.21 ± 3.93*	7.44 ± 3.53	6.93 ± 2.99
	AG	81.24 ± 20.65	84.67 ± 11.44	20.49 ± 8.02	22.17 ± 5.95	17.76 ± 5.73	19.81 ± 3.25	13.70 ± 3.53	14.35 ± 2.75	16.67 ± 5.22	15.85 ± 3.71*	7.58 ± 4.03	7.22 ± 3.03
	GG	84.45 ± 19.93	81.79 ± 10.21	19.83 ± 7.23	21. 09 ± 6.60	19.03 ± 5.53	18.74 ± 2.70	14.85 ± 3.39	13.35 ± 2.23	17.90 ± 5.20	14.87 ± 3.14*	7.33 ± 3.96	7.43 ± 3.13
rs2577074	AA	84.45 ± 19.93	83.16 ± 9.36	19.83 ± 7.23	22.14 ± 5.56	19.03 ± 5.53	19.30 ± 2.79	14.85 ± 3.39	14.11 ± 2.63	17.90 ± 5.20	15.95 ± 4.08	7.33 ± 3.96	6.50 ± 2.66
	AG	81.24 ± 20.65	85.75 ± 10.34	20.49 ± 8.02	21.99 ± 6.14	17.76 ± 5.73	19.59 ± 3.34	13.70 ± 3.53	14.51 ± 2.38	16.67 ± 5.22	16.17 ± 3.75	7.58 ± 4.03	7.62 ± 3.05
	GG	80.46 ± 18.15	84.48 ± 12.39	20.22 ± 6.60	22.54 ± 6.09	17.02 ± 5.45	20.17 ± 3.62	14.39 ± 2.97	13.63 ± 2.83	16.73 ± 5.62	16.21 ± 3.26	7.44 ± 3.53	7.04 ± 3.41
rs2249810	AA	82.33 ± 21.05	87.79 ± 14.62	18.87 ± 7.51	23.07 ± 6.58	18.80 ± 5.80	21.00 ± 4.28	15.03 ± 3.54	13.86 ± 3.03	17.70 ± 5.55	16.57 ± 3.82	6.80 ± 3.89	6.71 ± 3.43
	AG	81.67 ± 20.87	85.00 ± 10.52	20.46 ± 8.01	21.97 ± 6.19	17.85 ± 5.84	19.60 ± 3.03	13.66 ± 3.57	14.38 ± 2.53	16.88 ± 5.35	16.29 ± 3.54	7.68 ± 4.05	7.32 ± 3.19
	GG	81.77 ± 17.73	83.92 ± 9.27	20.74 ± 6.54	21.90 ± 5.56	17.42 ± 5.20	19.27 ± 3.12	14.42 ± 2.88	14.25 ± 2.48	16.74 ± 5.18	16.05 ± 3.92	7.57 ± 3.61	7.17 ± 2.83
rs2118343^a^	CC	82.74 ± 19.47	85.00 ± 2.00	21.04 ± 7.30	19.33 ± 0.58	17.71 ± 5.57	20.00 ± 3.46	14.38 ± 3.23	13.33 ± 2.52	17.23 ± 5.47	18.33 ± 3.51	7.43 ± 3.93	8.00 ± 4.00
	CG	80.05 ± 20.86	85.17 ± 10.43	19.03 ± 7.57	21.90 ± 5.72	17.90 ± 5.77	19.35 ± 3.29	13.63 ± 3.58	13.90 ± 2.41	16.63 ± 5.22	16.53 ± 3.45	7.68 ± 3.84	7.60 ± 3.21
	GG	85.43 ± 15.64	84.50 ± 10.63	20.71 ± 8.28	22.32 ± 6.09	20.14 ± 5.18	19.68 ± 3.21	15.71 ± 3.20	14.40 ± 2.61	16.71 ± 4.23	15.86 ± 3.88	6.43 ± 3.82	6.96 ± 2.92
rs6506891	AA	81.07 ± 20.23	84.08 ± 9.28	18.48 ± 7.34	22.06 ± 5.66	18.55 ± 5.74	19.31 ± 3.11	15.14 ± 3.55	14.27 ± 2.46	17.55 ± 5.59	15.95 ± 3.97	6.41 ± 3.33	7.17 ± 2.81
	AT	79.48 ± 20.69	84.84 ± 10.51	19.08 ± 7.65	21.93 ± 6.14	17.69 ± 5.61	19.63 ± 3.01	13.29 ± 3.56	14.34 ± 2.53	16.31 ± 4.84	16.20 ± 3.58	7.85 ± 3.92	7.32 ± 3.17
	TT	82.94 ± 19.02	86.87 ± 14.53	21.39 ± 7.24	23.20 ± 6.36	17.68 ± 5.60	20.67 ± 4.32	14.32 ± 3.15	13.67 ± 3.02	17.10 ± 5.49	16.40 ± 3.74	7.52 ± 3.86	6.53 ± 3.38

* *P* < 0.05, compared with each other genotype, LSD tests

^a^Combine stage 1 and 2 samples: rs2118343 genotypes were significantly associated with negative factor scores

## Discussion

This study investigated *CDNF2* mutations associated with SZ and associated symptoms in the Han Chinese population. Significant differences were found in genotype and allele frequencies of rs2249810 between SZ patients and healthy controls, suggesting that *CDNF2* is a susceptibility gene for SZ. We also found that rs2118343 genotypes are associated with negative psychotic symptoms of SZ.

*CDNF* has strong neuroprotective and restorative effects in animal models of PD [22], and protects dopaminergic neurons in the 6-hydroxydopamine rat model [14, 23]. However, the genotype and allele frequencies of the *CDNF* SNPs rs1901650 and rs11259365 did not differ between PD patients and controls; only the C allele of *CDNF* rs7094179, an intronic SNP, has been linked to PD susceptibility [24, 25].

Intron variant was relation to the *CDNF2* gene organization. *CDNF2* can protect and repair the dopaminergic system, and may thus have an important role in PD [14, 26, 27]. Five SNPs of *CDNF2* in our study were located in intron region of chromosome 10, which variation may lead to dopaminergic system disfunction. Meanwhile, multiple SZ susceptibility loci have been found on chromosome 10 [7, 8]. In stage 1 samples, we did not detect any associations in paranoid SZ patients, possibly because we did not examine other SZ subtypes. We therefore included the undifferentiated subtype in the stage 2 analysis, and found that rs2249810 of *CDNF2* may be a susceptibility locus in SZ.

SZ is influenced by dopamine, glutamate, and serotonin neurotransmission systems. We previously reported associations between Solute carrier family 6 member 4 [17] in the serotoninergic system and Glutamate ionotropic receptor NMDA type subunit 2B [16] and Reelin [28] in the glutamatergic system and SZ. The present study focused on *CDNF2* because of its role in the dopaminergic system. Our results from two sets of samples suggest that SNP rs2249810 of *CDNF2* may be a susceptibility locus for SZ and provide evidence that it is caused by the interaction of a large number of susceptibility genes [29]. To date, there has been a lot of GWAS researches on the susceptibility site of SZ [7–11, 30], and a recent meta-analysis using GWAS data also found 30 new sites [31]. However, there is still no reported association between *CDNF2* and SZ. The difference between GWAS and our research is samples selected more than three groups and multi-ethnic in the former, one group and only Han Chinese people in the latter. Therefore, our sample has a single genetic background. Meanwhile, we found rs2577075 genotypes and haplotype TGATC may be susceptibility factors of SZ in stage 2 samples, but those results were not found in total samples. That may be the majority of the samples were paranoid, and with less undifferentiated in total samples. Therefore, those results need to be further validated in a large sample, especially in the samples with multiple subtypes of SZ.

SZ is characterized by positive and negative symptoms, cognitive deficits, and disorganization of thoughts and behaviors [32, 33]. SZ is considered a dopamine disorder based on the psychosis-inducing effects of dopamine-releasing drugs such as amphetamines, and the anti-psychotic effects of drugs that block the dopamine D2 receptor [34]. Dopamine dysfunction has been identified as the major cause of SZ [35]. Our results have shown that *CDNF2* gene polymorphisms underlie the manifestations of SZ symptoms; moreover, rs2118343 genotypes were associated with negative factor subscores in SZ patients; this provides the first evidence of an association between *CDNF2* and negative symptoms in SZ and is consistent with our previous observations of a genetic basis for SZ symptoms [16, 17, 28].

This study had some limitations. Firstly, our sample size was not large enough to obtain complete PANSS scores. Secondly, although we validated our results in two independent datasets, undifferentiated subtypes were only included in the stage 2 analysis.

## Conclusion

In summary, our findings suggest that SNP rs2249810 of *CDNF2* is a novel susceptibility locus in SZ. Additional studies are needed to determine whether there are other SNPs associated with specific SZ subtypes (catatonic, collapse, and residual) and ethnic populations.

## Abbreviations

SZ: schizophrenia
CDNF: cerebral dopamine neurotrophic factor
BDNF: brain-derived neurotrophic factor
PD: Parkinson’s disease
SNPs: single nucleotide polymorphisms
DSM-IV: diagnostic and statistical manual of mental disorders fourth edition
OR: odds ratio
95% CI: 95% confidence intervals
LD: linkage disequilibrium
ANOVA: analysis of variance
PANSS: positive and negative symptom scale
